# Breastfeeding and complementary feeding in fragile settings: the case of Syrian refugees and their host communities in North Lebanon

**DOI:** 10.1186/s13006-022-00480-x

**Published:** 2022-05-14

**Authors:** Sara Daher, Fouad Ziade, Lara Nasreddine, Moomen Baroudi, Farah Naja

**Affiliations:** 1 Faculty of Public Health, Lebanese University, Tripoli, Lebanon; 2 Nutrition and Food Sciences Department, Faculty of Agriculture and Food Sciences, American University of Beirut, Riad El Solh, 1107 2020 Lebanon; 3 Department of Clinical Nutrition and Dietetics, College of Health Sciences, Research Institute of Medical & Health Sciences (RIMHS), University of Sharjah, Sharjah, 27272 UAE; 4 Faculty of Agriculture and Food Sciences, American University of Beirut, Riad El Solh, 1107 2020 Lebanon

**Keywords:** Breastfeeding, Complementary feeding, Fragile setting, Infant feeding, Lebanon, Host communities, Refugees

## Abstract

**Background:**

Adequate breastfeeding and complementary feeding practices are paramount in fragile situations where access to food and healthcare is limited. The objectives of this study are to examine breastfeeding and complementary feeding practices among Syrian refugees and their Lebanese host communities and to investigate the correlates of exclusive breastfeeding (EBF) at four and 6 months in these communities.

**Methods:**

Using two-stage stratified sampling, a cross-sectional survey was conducted in Akkar, a region with a high density of Syrian refugees in Lebanon, between April and November 2019. In one-to-one interviews, mothers of children (6–24 months) completed a questionnaire including specific questions about breastfeeding and complementary feeding practices, a 24-h recall, and socio-demographic characteristics for 189 Syrian refugees and 182 Lebanese host community households. Descriptive statistics, simple and multiple logistic regression were used in data analysis.

**Results:**

Among breastfeeding practices, ever-breastfeeding was most prevalent (90%), followed by early initiation of breastfeeding (64.8%), EBF at four (49.6%), and six (36%) months. One in four children was introduced to solids before 6 months of age, and less than a third was given iron-fortified baby cereals as the first complementary foods. Only 24.4% and 9.2% of children met the minimum dietary diversity and minimum acceptable diet requirements, respectively. Compared to children of the Lebanese host communities, those of Syrian refugees had higher rates of EBF at four and 6 months as well as continued breastfeeding at 1 year, whereas only 17.9% of Syrian refugees’ children met minimum dietary diversity compared to 30.9% of Lebanese host community children (*p* <  0.05). Among refugees, education and spouse’s employment status were associated with higher odds of EBF at 4 months. As for Lebanese households, female children were less likely to be exclusively breastfed at 4 months and 6 months, while a natural delivery increased the odds of EBF at 6 months.

**Conclusion:**

Breastfeeding and complementary feeding practices are suboptimal among children of Syrian refugees and their Lebanese host communities in North Lebanon. There is a need for intervention strategies to tackle gaps in services and assistance delivery programs to enhance infant and young child feeding practices among both communities.

## Background

During the first 1000 days of life, adequate breastfeeding and complementary feeding are critical to ensure optimal growth, development, and health later in life [1]. The health advantages of breastfeeding seem to be most critical in fragile settings, such as those created by conflicts, epidemics, and natural disasters. In such settings, people are forced to live in unsanitary, crowded conditions with limited access to food and health care, and at high risk of infection with diarrheal diseases [2]. Breastfeeding provides an accessible, nutritious, and safe food source for infants and toddlers [3] and better health for women [4, 5]. It is important to note, however, that in many instances, the situations of fragility are accompanied by crowded settings, traumatic stress experiences, physical and emotional struggles, all of which could reduce the supply of breast milk and may hamper the ability to breastfeed.

The Syrian refugee crisis constitutes a recent case of such a fragile situation. The displacement caused by the Syrian war has been the largest reported second only to that reported after World War II [6]. Since its start in 2011, the Syrian war has led to the massive, protracted displacement of more than 6.5 million refugees, with the majority fleeing to neighboring countries such as Lebanon [7, 8]. The arrival of these large numbers of refugees contributed to a situation of unsustainable and rapid urbanization in Lebanon [9]. This is particularly true in areas such as Akkar, one of the largest and most marginalized districts in Lebanon, with more than 41% of its Lebanese residents considered deprived in 2018 [10]. Due to its proximity to the Lebanese-Syrian borders, Akkar was estimated to host more than one third of the district’s population [11]. This rise in population density has added pressure to the already fragile infrastructure and under-served basic services in Akkar [12].

Despite the importance of breastfeeding and complementary feeding in fragile settings, no studies have addressed them among Syrian refugees and their host communities [13]. The main objective of this study is to examine breastfeeding and complementary feeding practices among children aged 6–24 months old belonging to households of Syrian refugees and their Lebanese host communities in Akkar, North of Lebanon. A secondary objective was to examine the correlates of exclusive breastfeeding (EBF) at four and 6 months in the study population. Findings of this study provided the evidence base for the development of intervention strategies to tackle gaps in feeding practices, improve services and assistance delivery programs to enhance infant and young child feeding practices among both communities.

## Methods

### Study design

This is a cross-sectional study that was conducted in Akkar, a rural district in North Lebanon. A two-stage sampling technique was used to select the households of Syrian refugees and their Lebanese host communities. Households were considered as the sampling unit. Villages in Akkar were considered as the strata. In Akkar, there are 293 villages and towns, 217 of which are currently hosting Syrian refugees following the conflict in Syria [14]. Of these villages, only those with at least 2500 registered Syrian refugees were selected as these villages were considered to host a high number of refugees by the United Nations High Commissioner for Refugees (UNHCR) [15]. These villages were Aamayer, Mhammaret, Bbenine, Jabal Akroum, Biret Akkar, Berqayel, and Qobbet Chamra.

Within each stratum (village), a systematic sampling approach was followed to select the households (every tenth household). The number of households in each village was proportional to the population density of this village. In the village, households were invited to participate if they had a mother and a child aged between 6 and 24 months. Additional inclusion criteria were: 1) Mothers and children within each of the households were both of Lebanese nationality (holder of a Lebanese identification card) or both of Syrian nationality (holder of a Syrian identification card) and 2) mothers and their participating children were living in the household. In the case where there was more than one child between the ages of 6–24 months in the household, the younger child was selected since the main interest in this study is the breastfeeding and feeding practices among children, and mothers tend to remember more clearly data of the younger child. Sample size calculations were based on a 27.4% prevalence of EBF at 6 months, as reported in an earlier investigation in Lebanon in 2013 [16]. Using a confidence level of 95% and a margin of error of 5%, the sample size needed for the study was 306 households [17]. Accounting for a dropout / refusal rate of 16%, the sample size was increased to 370 households.

The Institutional Review Board of the Social and Behavioral Sciences at the Lebanese University reviewed and approved the protocol of this study. Key stakeholders and municipality officials from each of the identified villages were contacted and their approval was obtained prior to any field visits.

Trained field surveyors approached mothers and children within their household setting and explained the purpose and procedure of the study using a consent form. The interviewers emphasized that participation was completely voluntary. Participants were informed that they could withdraw at any time without any penalties.

### Data collection

Data collection took place between April and November 2019. In the household, data collection was conducted by a one-to-one interview which lasted around 35 min and involved the completion of a multi-component questionnaire and obtaining anthropometric measurements of the mother. The questionnaire consisted of the following sections: socio-demographic characteristics of the mother, basic characteristics of the child, a 24-h recall for the child as well as breastfeeding and complementary feeding practices.

The socio-demographic data included mother’s characteristics (age, educational level, employment status, and smoking status), spouse’s characteristics (educational level and employment status), in addition to household’s monthly income, and crowding index.

The child data included age, gender, and birth method (natural birth or Cesarean section).

Dietary intake was assessed by 24-h recall using the Multiple Pass Food Recall 5-step approach as developed by the United States Department of Agriculture. This approach consisted of five steps: 1) a quick food list recall, 2) forgotten food list probe 3) time and occasion at which foods were consumed, 4) detailed overall cycle, and 5) final probe review of the foods consumed [18]. Common household measures (measuring cups, spoons, and a ruler) were shown to assist in estimation of portion size [19].

The breastfeeding and complementary feeding practices were examined through the following questions in the questionnaire:

- *Child was ever breastfed*: proportion of children ever breastfed no matter the duration.
- *Early initiation of breastfeeding*: proportion of children who were put to the breast within 1 hour of birth.
- *EBF for four and 6 months*: proportion of children who were exclusively breastfed for four and 6 months. It was explained to the mothers that EBF is defined as infant receiving only breast milk (from the breast or as expressed milk) with no other liquids (including water and traditional medicines such as teas and herbal infusions) and solid foods, with the exception of oral rehydration solution and drops / syrups of vitamins, minerals, or medications [20].
- *Continued breastfeeding for 1 year*: proportion of children who continued breastfeeding until 1 year.
- *Mean duration of EBF*: number of months during which the child was exclusively breastfed.
- *Mean duration of breastfeeding*: number of months during which the child was breastfed.
- *Primary reasons for initiating breastfeeding:* mothers were given a list of reasons to choose from. The list included the following: health benefits for mother; health benefits for baby; previous breastfeeding experiences encouraged it further; high formula cost; doctor’s advice; and family advice.
- *Primary reasons for not breastfeeding and stopping breastfeeding*: mothers were given a list of commonly reported reasons and were asked to select those that were relevant to their situations. In case the reason they have experienced was not included within the list, they had the option to add it.
- *Infants used formula before one and 3 months*: proportion of children who were offered infant formula before one and 3 months of age. Starting infant formula included introducing infant formula in addition to or without breastfeeding.
- *Mothers introduced solids or semi-solids before* *four and* 6 *months*: proportion of children who were introduced to solid or semi-solid food before four and 6 months of age. Solid or semi-solid food introduction was defined as starting solids or semi-solid food in addition to breast milk or infant formula.
- *Mean age of solid food introduction*: child age when solid foods were introduced (in months).
- *Reasons to start solid food introduction*: mothers were given a list and were asked to select the most relevant option.
- *Types of first food introduced*: mothers were asked about the name of the first solid / semi-solid food child was introduced to.
- *Mean age of introduction of each food group*: average age of food introduction (in months).
- *Minimum dietary diversity (MDD)*: based on dietary intakes collected using the 24-h recalls, proportion of children aged 6–23.9 months who consumed foods from at least five out of eight groups. These groups included: breast milk; grains, roots, and tubers; legumes and nuts; dairy products; flesh foods; eggs; vitamin A rich fruits and vegetables, and other fruits and vegetables. MDD was also calculated using the older definition, where breast milk was not included as a food group: consuming foods from at least four out of seven groups [21, 22].
- *Minimum acceptable diet (MAD)*: a composite indicator for breastfed and non-breastfed children based on MDD and minimum meal frequency (MMF is defined as the proportion of children 6–23.9 months of age who receive solid, semi-solid, or soft foods (including milk feeds for non-breastfed children) the minimum number of times or more. The minimum number was defined as: two times for breastfed infants 6–8 months; three times for breastfed children 9–23.9 months; four times for non-breastfed children 6–23.9 months in the previous day). MAD was met in the following conditions:
  *○ Breastfed children*: children 6–23.9 months of age who had both MDD and MMF.
  *○ Non-breastfed children*: children 6–23.9 months of age who had both MDD and MMF in addition to at least two milk feedings.
  MAD was calculated using the two definitions of MDD [21, 22].

Mother’s weight and height were measured using calibrated equipment and standard techniques. Body mass index (BMI) was calculated by dividing weight by height squared (kg / m2) [23].

The questionnaire was completed by the field surveyors and anthropometric measures were taken at the end of the interview. The questionnaire was pilot-tested on 20 mothers (10 Lebanese and 10 Syrian refugees) and data were discarded. The results of the pilot-testing were used to improve the cultural and social acceptability of the questionnaire. More specifically, reasons for not breastfeeding and stopping breastfeeding were adjusted to include additional options mentioned by mothers that were not included in the original version of the questionnaire (such as sense of embarrassment and mother didn’t like breastfeeding). Field surveyors utilized calibrated scales and standardized techniques in order to minimize any intra-interviewer and inter-interviewer bias.

### Statistical analysis

Data were entered and analyzed using Statistical Package for the Social Sciences (SPSS) program (version 21.0). Descriptive statistics were performed and presented as means and standard deviations (SD) for the continuous variables and as frequencies and percentages for the categorical ones. The difference in socio-demographic characteristics, child data, and mothers’ characteristics between the Lebanese host communities and Syrian refugees was examined using chi-square tests for categorical variables and independent t-tests for continuous variables.

Simple binary logistic regression was used to investigate the relationship between explanatory variables and EBF at four and at 6 months. Results are reported as crude odds ratios (OR) with 95% confidence intervals (CI). For the multiple regression, variables that were significant (*p* <  0.2) in the bivariate analysis were included. A *P*-value lower than 0.05 was considered significant.

## Results

Out of 407 eligible households, a total of 371 households agreed to participate in this study (response rate 91.2%). The two most commonly cited reasons for refusal to participate were lack of time and being not convinced of the utility of the research objectives.

Table 1 shows the descriptive characteristics of the study population. A larger proportion of Syrian refugee mothers were younger than 30 years as compared with Lebanese mothers (70.9% vs 45.6%), had an educational level of primary school or less (55.3% vs 23.8%), were non-smokers (84.7% vs 55.5%), and were underweight / normal weight (46.2% vs 30.9%). As for the children, 40.7% of children were aged 6–12 months and 59.3% were aged 13–24 months, 54.2% were males, and 27.9% were born by Cesarean section. Significantly more children of Lebanese host communities were born by Cesarean section compared with Syrian refugees’ children (35.6% vs 20.6% respectively). Furthermore, 40.6% of households had a monthly income of less than $200, 70.1% had a crowding index of two or more individuals per room, 50% of the spouses had an educational level of primary school or less, and 81.3% of the spouses were employed. Significant differences were observed between Lebanese host communities and Syrian refugee households at the level of socio-economic characteristics.

**Table 1:** Descriptive characteristics of the study population (*n* = 371)

	Total sample (***n*** = 371)	Syrian refugees (***n*** = 189)	Lebanese host communities (***n*** = 182)	***P***-value^*^
***Mothers’ characteristics***				
Age (years)				
< 30	209 (58.2)	127 (70.9)	82 (45.6)	**< 0.001**
≥ 30	150 (41.8)	52 (29.1)	98 (54.4)	
Educational level				
Primary school or less	147 (39.8)	104 (55.3)	43 (23.8)	**< 0.001**
Intermediate school or above	222 (60.2)	84 (44.7)	138 (76.2)	
Employment status				
Not employed	335 (91.0)	171 (91.9)	164 (90.1)	0.540
Employed	33 (9.0)	15 (8.1)	18 (9.9)	
Smoking status				
Non-smoker (including past smoker)	261 (70.4)	160 (84.7)	101 (55.5)	**< 0.001**
Current smoker	110 (29.6)	29 (15.3)	81 (44.5)	
Nutritional status **				
Underweight / normal weight (BMI < 25 kg / m^2^)	142 (38.7)	86 (46.2)	56 (30.9)	**0.003**
Overweight / obese (BMI ≥ 30 kg/m^2)^	225 (61.3)	100 (53.8)	125 (69.1)	
***Child’s characteristics***				
Age (months)				
6–12 months	151 (40.7)	79 (41.8)	72 (39.6)	0.661
13–24 months	220 (59.3)	110 (58.2)	110 (60.4)	
Gender				
Male	201 (54.2)	96 (50.8)	105 (57.7)	0.182
Female	170 (45.8)	93 (49.2)	77 (42.3)	
Birth method				
Cesarean section	103 (27.9)	39 (20.6)	64 (35.6)	**0.001**
Natural birth	266 (72.1)	150 (79.4)	116 (64.4)	
***Socio-economic characteristics***				
Monthly income				
Less than $200	146 (40.6)	123 (68.7)	23 (12.7)	**< 0.001**
More than $200	214 (59.4)	56 (31.3)	158 (87.3)	
Crowding index				
< 2 individuals per room	110 (29.9)	10 (5.3)	100 (55.2)	**< 0.001**
≥ 2 individuals per room	258 (70.1)	177 (94.7)	81 (44.8)	
Spouse’s educational level				
Primary school or less	188 (50.8)	119 (63.3)	69 (37.9)	**< 0.001**
Intermediate school or above	182 (49.2)	69 (36.7)	113 (62.1)	
Spouse’s employment status				
Not employed	68 (18.7)	57 (30.8)	11 (6.2)	**< 0.001**
Employed	295 (81.3)	128 (69.2)	167 (93.8)	

^*^P-values were derived from Chi-square for categorical variables and independent t-test for continuous variables

^**^BMI refers to body mass index, it was calculated by dividing weight by height squared (kg / m2)

**Fig. 1:**
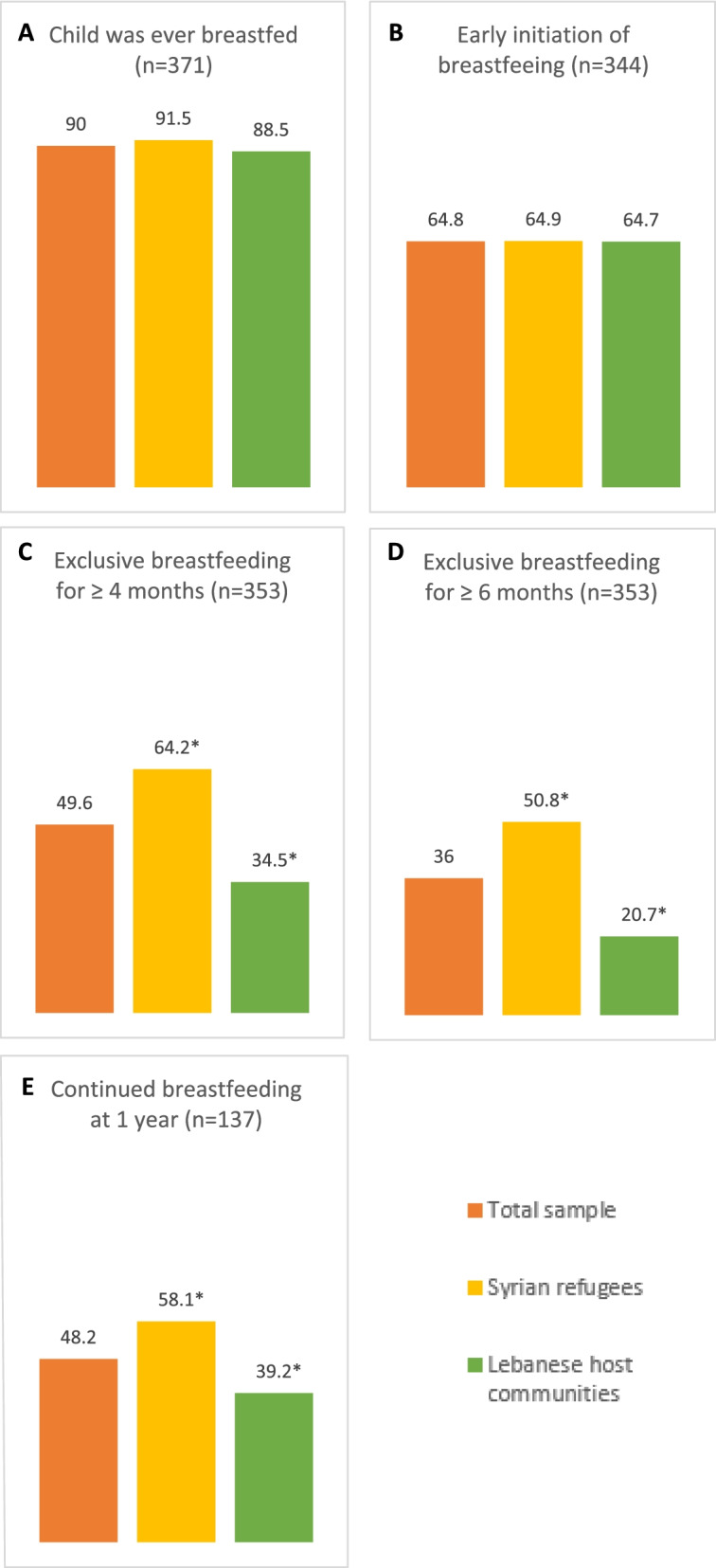
Breastfeeding characteristics of children 6–24 months among the study groups

Figure 1 shows the breastfeeding characteristics of the study population. Although the majority of the children (90%) were ever-breastfed, only 64.8% initiated breastfeeding early (within 1 hour of birth). Almost half of the children were exclusively breastfed for 4 months and continued breastfeeding until 1 year (49.6% and 48.2% respectively), whereas only 36% of children were exclusively breastfed for 6 months. The proportions of children who were exclusively breastfed for four and 6 months were significantly higher among children of Syrian refugees compared with children from Lebanese host communities (4 months: 64.2% vs 34.5%; 6 months: 50.8% vs 20.7% respectively). In addition, the rate of continued breastfeeding at 1 year was significantly higher among Syrian refugees’ children compared with Lebanese host communities’ children (67.6% vs 48.6% respectively).

The mean duration of EBF and breastfeeding were 3.5 ± 3.3 and 8.1 ± 7.3 months respectively as shown in Table 2. The mean duration of EBF and breastfeeding were significantly higher among Syrian refugees’ children compared with Lebanese host communities’ children (4.5 ± 3.1 vs 2.6 ± 3.1 and 10.0 ± 7.6 vs 6.5 ± 6.7 months respectively). The proportion of children who used infant formula before 1 month and 3 months were 32.1% and 45.0% respectively. Significantly more Lebanese children had infant formula before one and 3 months compared with Syrian refugees’ children (41.6% vs 23.2% and 62.4% vs 28.6% respectively). In addition, 4.6% and 25.9% of mothers introduced solid or semi-solid foods to their child before four and 6 months respectively, with a mean age of solid food introduction of 6.4 ± 2.3 months. The primary reason for starting solid food introduction was that the child was ‘still hungry after milk feeds’ (45.2%). Significantly more mothers from Lebanese host communities introduced solid or semi-solid foods to their child before 6 months compared with Syrian refugee mothers (31.4% vs 20.7% respectively), and the mean age of solid or semi-solid food introduction was significantly higher among Syrian refugees’ children compared with children from Lebanese host communities (6.7 ± 2.4 vs 6.0 ± 2.0 months respectively). The MDD and MAD rates are also shown in Table 2. When using the seven food groups’ definition, 36.4% and 10.5% of children met the MDD and the MAD respectively. Whereas when using the 8 food groups’ definition, only 24.4% and 9.2% of children met the MDD and MAD respectively. Furthermore, using either of the two definitions of MDD, significantly more children from Lebanese host communities met the MDD as compared with Syrian refugees’ children.

**Table 2:** Breastfeeding, infant formula feeding, and solid feeding characteristics of the study population

	Total sample	Syrian sefugees	Lebanese host communities	***P***-value
**Mean duration of EBF (months) (*n*** **= 353)**				
	3.5 ± 3.3	4.5 ± 3.1	2.6 ± 3.1	**< 0.001**
**Mean duration of breastfeeding (months) (*n*** **= 233)**				
	8.1 ± 7.3	10.0 ± 7.6	6.5 ± 6.7	**< 0.001**
**Primary reasons for initiating breastfeeding**^a^**(*n*** **= 322)**				
Health benefits for the baby	193 (59.9)	98 (58.7)	95 (61.3)	0.070
Health benefits for the mother	94 (29.2)	48 (28.7)	46 (29.7)	
High formula cost	7 (2.2)	7 (4.2)	0 (0.0)	
Others^b^	28 (8.7)	14 (8.4)	14 (9.4)	
**Primary reasons for not breastfeeding**^a^**(*n*** **= 36)**				
Problems with milk production	17 (47.2)	7 (46.7)	10 (47.6)	0.218
Pain or discomfort when breastfeeding	6 (16.7)	2 (13.3)	4 (19.0)	
Medical problems (Cesarean section, diabetes, etc)	5 (13.9)	4 (26.7)	1 (4.8)	
Mother was sick	4 (11.1)	2 (13.3)	2 (9.5)	
Others^b^	4 (11.1)	0 (0.0)	4 (19.0)	
**Primary reasons for stopping breastfeeding (*n*** **= 203)**				
Problems with milk production	44 (21.7)	14 (14.9)	30 (27.5)	**< 0.001**
Pain or discomfort when breastfeeding	43 (21.2)	20 (21.3)	23 (21.1)	
Subsequent pregnancy	36 (17.7)	32 (34.0)	4 (3.7)	
Baby didn’t accept the breast	18 (8.9)	4 (4.3)	14 (12.8)	
Needed help with feeding the baby	18 (8.9)	10 (10.6)	8 (7.3)	
Others^b^	44 (21.7)	14 (14.9)	30 (27.5)	
**Proportion of children who used infant formula before 1 month (*n*** **= 358)**				
	115 (32.1)	43 (23.2)	72 (41.6)	**< 0.001**
**Proportion of children who used infant formula before 3 months**^a^**(*n*** **= 358)**				
	161 (45.0)	53 (28.6)	108 (62.4)	**< 0.001**
**Proportion of mothers introducing solid or semi-solid foods before 4 months (*n*** **= 351)**				
	16 (4.6)	8 (4.5)	8 (4.7)	0.935
**Proportion of mothers introducing solid or semi-solid foods before 6 months (*n*** **= 351)**				
	91 (25.9)	37 (20.7)	54 (31.4)	**0.022**
**Mean age of solid or semi-solid food introduction (in months) (*n*** **= 351)**				
	6.4 ± 2.3	6.7 ± 2.4	6.0 ± 2.0	**0.003**
**Reasons to start solid-food introduction (*n*** **= 361)**				
Child was still hungry after milk feeds	163 (45.2)	86 (46.5)	77 (43.8)	0.138
Child was old enough	98 (27.1)	41 (22.2)	57 (32.4)	
Tradition in family	42 (11.6)	25 (13.5)	17 (9.7)	
Others^b^	58 (16.1)	33 (17.8)	25 (14.2)	
**Minimum dietary diversity (7 groups)**^c^				
No	232 (63.6)	133 (72.3)	99 (54.7)	**< 0.001**
Yes	133 (36.4)	51 (27.7)	82 (45.3)	
**Minimum acceptable diet (7 groups)**^c^				
No	323 (89.5)	164 (90.1)	159 (88.8)	0.691
Yes	38 (10.5)	18 (9.9)	20 (11.2)	
**Minimum dietary diversity (8 groups)**^d^				
No	276 (75.6)	151 (82.1)	125 (69.1)	**0.004**
Yes	89 (24.4)	33 (17.9)	56 (30.9)	
**Minimum acceptable diet (8 groups)**^d^				
No	324 (90.8)	163 (91.6)	161 (89.9)	0.595
Yes	33 (9.2)	15 (8.4)	18 (10.1)	

*EBF* exclusive breastfeeding

^a^ Cells have expected count less than 5 – used Fisher’s test instead of Pearson Chi-Square

^b^Other reasons are mentioned in the methodology section

^c^Based on the 24 h recall [21] (WHO, 2008)

^d^Based on the 24 h recall [22] (WHO, 2017)

**Fig. 2:**
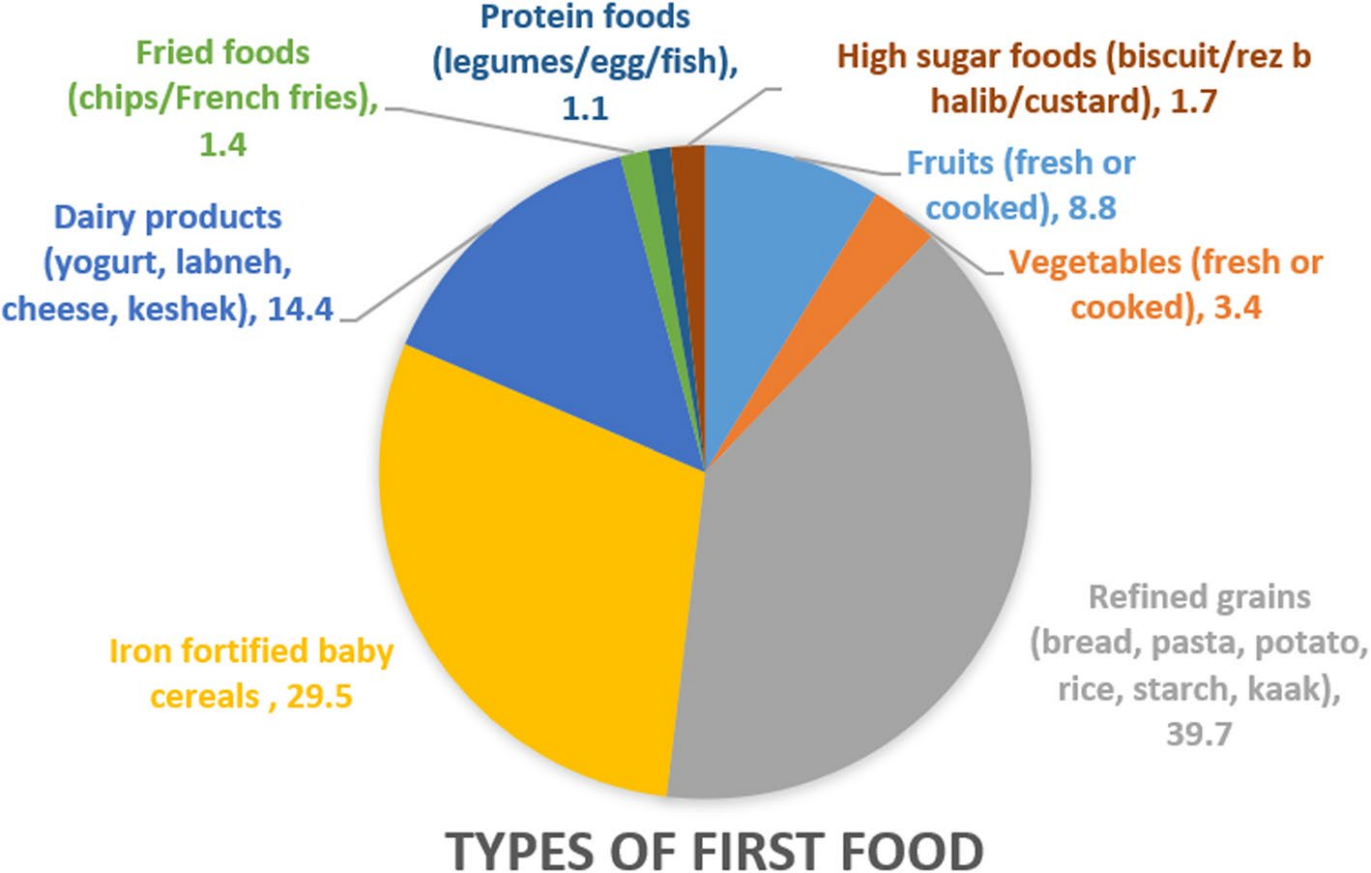
Types of first food introduced among the study population (*n* = 353)

As shown in Fig. 2 and Table 3, refined grains – such as bread, pasta, potato, rice, starch, and kaak – were reported as the most frequently consumed first foods among the study sample’s children (39.7%), introduced at a mean age of 6.7 ± 2.5 months, followed by iron-fortified baby cereals (29.5%) at 5.8 ± 1.4 months, dairy products – such as yogurt, labneh, cheese, keshek – (14.4%) at 6.6 ± 2.6 months, fruits – fresh or cooked – (8.8%) at 6.5 ± 2.0 months, vegetables – fresh or cooked – (3.4%) at 4.6 ± 1.2 months, high sugar foods – such as biscuits, rez b halib (rice pudding), and custard – (1.7%) at 5.8 ± 0.4 months, fried foods – such as chips and French fries – (1.4%) at 11.0 ± 2.2 months, and protein foods – such as legumes, egg, and fish – (1.1%) at 8.5 ± 2.5 months. Except for protein foods group, sizeable proportion of children received many food groups earlier than their corresponding recommended age of introduction: refined grains (21.5%); iron-fortified baby cereals (28.8%); dairy products (29.2%); fruits (25.8%); vegetables (75%); high sugar foods and fried foods (100%).

**Table 3:** Mean age and percentage of introducing first foods (in months) among study population children (*n* = 353)

Type of food	mean ± SD	Recommended age of introduction^a^	% Children introduced to food not according to recommendations
Refined grains	6.7 ± 2.5	6 months	21.5%
Iron-fortified baby cereals	5.8 ± 1.4	6 months	28.8%
Dairy products	6.6 ± 2.6	6 months (except cow’s milk)	29.2%
Fruits	6.5 ± 2.0	6 months	25.8%
Vegetables	4.6 ± 1.2	6 months	75%
High sugar foods [7]	5.8 ± 0.4	As late as possible (not before 24 months)	100%
Fried foods	11.0 ± 2.2	As late as possible	100%
Protein foods	8.5 ± 2.5	6 months	0%

^a^Recommendations were derived from the European Food Safety Authority, 2019 [24] and the American Academy of Pediatrics, 2020 [25]

The factors associated with EBF for more than four and 6 months among children of Lebanese host communities and Syrian refugees were analyzed using simple and multiple binary logistic regression (Tables 4 and 5).

**Table 4:** Correlates of EBF at 4 and 6 months among Syrian refugee households

Variable	EBF for 4 months or more		EBF for 6 months or more	
	OR	95% CI	OR	95% CI
**Mother’s age**				
< 30 years	1		1	
≥ 30 years	0.674	0.337, 1.346	0.932	0.473, 1.835
**Mother’s education**				
Primary school or less	1		1	
Intermediate school or above	1.175	0.631, 2.187	1.011	0.559, 1.829
**Mother’s employment status**				
Unemployed	1		1	
Employed	1.549	0.472, 5.084	1.101	0.381, 3.179
**Spouse’s educational level**				
Primary school or less	1		1	
Intermediate school or above	***2.552***	1.284, 5.071	1.356	0.735, 2.503
**Spouse’s employment status**				
Not employed	1		1	
Employed	***3.164***	1.627, 6.152	*2.368*	1.226, 4.571
**Crowding index**				
< 2 person per room	1		1	
≥ 2 person per room	0.745	0.186, 2.986	1.012	0.282, 3.626
**Monthly income**				
Less than $200	1		1	
More than $200	1.104	0.557, 2.185	1.120	0.584, 2.147
**Child gender**				
Male	1		1	
Female	1.317	0.713, 2.431	0.978	0.544, 1.758
**Birth method**				
Cesarean section	1		1	
Natural birth	*1.619*	0.781, 3.356	1.562	0.758, 3.223
**Time after delivery put to breast**				
≤ 1 h	1		1	
> 1 h	*0.560*	0.280, 1.119	*0.543*	0.282, 1.045
**Mother is a current smoker**				
No	1		1	
Yes	0.937	0.401, 2.189	0.619	0.270, 1.421
**Mother’s nutritional status**				
Underweight / normal weight	1		1	
Overweight / obese	0.735	0.394, 1.370	0.739	0.408, 1.336

*EBF* exclusive breastfeeding, *CI* confidence interval, *OR* odds ratio

Italics-style OR are significant at a *p* < 0.2 in simple binary regression

Bolded OR are significant at *p* < 0.05 in multiple binary regression

**Table 5:** Correlates of EBF at 4 and 6 months among children from Lebanese host communities

Variable	EBF for 4 months or more		EBF for 6 months or more	
	OR	95% CI	OR	95% CI
**Mother’s age**				
< 30 years	1		1	
≥ 30 years	1.383	0.731, 2.615	*1.835*	0.846, 3.979
**Mother’s education**				
Primary school or less	1		1	
Intermediate school or above	1.336	0.624, 2.861	1.368	0.549, 3.404
**Mother’s employment status**				
Unemployed	1		1	
Employed	1.156	0.399, 3.349	1.312	0.397, 4.341
**Spouse’s educational level**				
Primary school or less	1		1	
Intermediate school or above	1.049	0.552, 1.993	1.010	0.476, 2.143
**Spouse’s employment status**				
Not employed	1		1	
Employed	2.447	0.511, 11.717	2.823	0.349,22.811
**Crowding index**				
< 2 person per room	1		1	
≥ 2 person per room	*1.548*	0.825, 2.905	1.210	0.580, 2.522
**Monthly income**				
Less than $200	1		1	
More than $200	1.249	0.483, 3.227	1.880	0.526, 6.719
**Child gender**				
Male	1		1	
Female	***0.480***	0.249, 0.925	***0.200***	0.078, 0.511
**Birth method**				
Cesarean section	1		1	
Natural birth	1.293	0.665, 2.515	***1.823***	0.793, 4.190
**Time after delivery put to breast**				
≤ 1 h	1		1	
> 1 h	1.017	0.524, 1.972	0.983	0.455, 2.125
**Mother is a current smoker**				
No	1		1	
Yes	1.120	0.598, 2.098	1.989	0.945, 4.187
**Mother’s nutritional status**				1
Underweight / normal weight	1		1	
Overweight / obese	0.682	0.351, 1.328	0.756	0.349, 1.635

*EBF* exclusive breastfeeding, *CI* confidence interval, *OR* odds ratio

Italian-style OR are significant at a *p*-value < 0.2 in simple binary regression

Bolded OR are significant at *p* < 0.05 in multiple binary regression

The analysis showed that the spouse’s educational level, spouse’s employment status, child’s birth method, and early initiation of breastfeeding were factors associated significantly with EBF for more than 4 months among Syrian refugees (Table 4). After adjustment, a higher educational level of the spouse and his employment status remained significant correlates of EBF at 4 months (OR: 3.11; 95% CI: 1.40, 7.16; OR: 3.37; 95% CI: 1.54, 7.37, respectively) (data not shown). On the other hand, although employment status of the spouse and early initiation of breastfeeding were significant predictors of EBF at 6 months among Syrian refugees (Table 4), none remained significant after adjustment (data not shown).

Crowding index and the gender of the baby were associated with EBF at 4 months among children of Lebanese host communities (Table 5). After adjustment, only the female sex of the child remained significantly associated with lower odds of EBF at 4 months (OR: 0.49; 95% CI: 0.25, 0.95) (data not shown). At 6 months, the factors that showed significant associations with EBF included: age of the mother, gender of the child, and child’s birth method (Table 5). The results of the multiple regression showed that, after adjustment, both the gender of the child (female) and the birth method remained significant correlates of EBF at 6 months (OR: 0.17; 95% CI: 0.06, 0.47 and OR: 2.54; 95% CI: 1.02, 6.33, respectively) (data not shown).

## Discussion

This study is the first to examine infant and young child feeding practices among Syrian refugees and their Lebanese host communities. The findings showed suboptimal breastfeeding and complementary feeding practices among both study groups. Distinct determinants for EBF at four and 6 months among children of the Syrian refugees and those of Lebanese host communities were revealed.

The rates of early initiation of breastfeeding observed among both study groups (64.9% and 64.7% among Syrian refugees and Lebanese host communities, respectively) were similar to those observed among Turkish households and Syrian refugees in Turkey (71.1% and 61.4% respectively [26]). These rates were higher than those reported globally in 2017 (44% [27]), in the Middle East and North Africa (MENA) region in 2017 (34.3% [28]), and among Syrian refugees in Jordan in 2016 (37.1% [29]). The rural nature of this study location may explain the higher rates of early initiation of breastfeeding, as traditions encourage mothers to breastfeed early and, unless there are any medical complications, mothers are expected to take care of their newborn immediately after birth.

Concerning EBF at 6 months, results differed among the two groups studied, whereby households of Lebanese host communities reported lower rates as compared to those of Syrian refugees: 20.7% vs 50.8% respectively. The rate of EBF at 6 months among Lebanese host communities in this study (20.7%) is comparable to that of the MENA region (20.5%) [28], however it is lower than rates globally (40%) [27], in Turkey in 2020 (34.1% [26]), in the United Arab Emirates (UAE) in 2013 (25% [30]) and in Jordan in 2017 (33% [31]). As for Syrian refugees’ children, their rate of EBF at 6 months (50.8%) was higher than that reported for Syrian refugees in Turkey in 2020 (28.1% [26]) and Syrian refugees in Jordan in 2016 (19.1% [31]). The dire circumstances Syrian refugees in Akkar are facing in addition to their refugee situation may have contributed to their increased dependence on breastfeeding as the main nutrition source for their infants. On the other hand, the low rates observed among Lebanese host communities in Akkar can be attributed firstly to the perception of problems with milk production as 27.5% of Lebanese mothers reported problems with milk production as the primary reason for stopping breastfeeding, and secondly to the higher incidence of Cesarean section birth among Lebanese host communities compared with Syrian refugees in this study (35.6% vs 20.6% respectively). Cesarean section birth was reported by previous studies to be associated with a lower incidence of breastfeeding [32, 33].

Furthermore, rates of continued breastfeeding at 1 year among Lebanese host communities in this study (39.2%) are similar to those found in an earlier investigation in Lebanon in 2005 (35% [34]) and the UAE (37% [35]). These rates are however lower compared with rates globally (74% ([27] and in Turkey in 2020 (63.8% [26]). Among Syrian refugees, the rates of continued breastfeeding at 1 year (58.1%) were similar to rates among Syrian refugees in Jordan and Turkey (56.5% and 55.9% respectively [26, 31]).

Taken together, the rates related to the indicators of breastfeeding in this study indicated higher mean duration of breastfeeding and EBF among Syrian refugee households compared with households of Lebanese host communities. Lebanese households may have had the option of transferring to infant formula feeding or early introduction of solid foods and stopping breastfeeding or EBF, whereas Syrian refugee households may find it more difficult to procure foods and thus depend on breastfeeding for a longer period as the source of child nutrition. This is also seen by the lower mean age of solid or semi-solid food introduction among households of Lebanese host communities compared with Syrian refugee households indicating later initiation of complementary feeding among Syrian refugee households thus longer periods of breastfeeding and EBF.

The American Academy of Pediatrics recommends that infants not be introduced to solid foods before the age of 6 months as younger children (especially those aged less than 4 months) are not developmentally ready for solid foods [36]. Existing evidence indicated that early introduction of solids may increase the risk of some chronic diseases, such as obesity, celiac disease, eczema, and diabetes [37]. Results from this study showed that around one quarter of mothers introduced solid or semi-solid foods to their children before 6 months of age, with higher rates among households of Lebanese host communities compared with Syrian refugee households (31.4% vs 20.7% respectively). Rates of children from Lebanese host communities who were introduced to solid foods before 6 months were similar to rates globally (29%) and in the MENA region (27%) [38]. However, these rates fall below earlier estimates from Lebanon in 2010 (74.8%) [39], in Jordan in 2019 (54.3%) [40]. Reasons for the introduction of solid foods reported in this study included that the child was still hungry after milk feeds, child was old enough, and tradition in family. All these reasons are subjective and may indicate gaps of knowledge among mothers on when to appropriately initiate complementary feeding for children. Such reasons were also commonly observed in other studies in Jordan, the UAE, and the United States of America (USA) [30, 40].

Furthermore, the World Health Organization (WHO) recommends the introduction of traditional iron-fortified cereal and meat as the first complementary foods to meet the iron requirements of growing infants and decrease the risk of iron deficiency [41]. However, less than one third (29.5%) of infants in the study sample were given iron-fortified baby cereals and only 1.1% were given protein foods (iron rich foods), while 40% were given unfortified refined grains and 8.8% and 3.4% were given fruits and vegetables respectively. In addition, fried foods and high sugar foods were also reported as first foods introduced among 3.1% of infants in the study sample. The observed gap between recommendations and actual suboptimal complementary feeding practices observed in this study sample can be attributed to both food availability problems in these fragile communities as well as poor knowledge about feeding practices. The lack of knowledge about complementary feeding practices among Syrian refugee mothers in Lebanon has been previously documented and was shown to affect not only types of solid foods, but also quantities and timely initiation of solid foods [42]. These findings indicated the need for promoting infant feeding practices, not only in terms of providing financial aids but also educational programs and awareness campaigns to spread appropriate complementary feeding practices.

The MDD rates were higher among children of Lebanese host communities (45%) compared with Syrian refugees’ children (27%). These lower rates are understandable in light of the lower availability of food items among Syrian refugees and later introduction of solid foods as observed in this study. In addition, the MAD rates were extremely low among both study groups’ households (11.2% and 9.9% among Lebanese host communities and Syrian refugee households respectively). These two indicators show that dietary diversity is poor during the complementary feeding period, though poorer among Syrian refugee households. This is especially concerning, as poor dietary diversity can put infants and young children at an increased risk of inadequate intake of various essential micronutrients, especially zinc and iron [43], and may be associated with stunting as shown in many studies from low- and middle-income countries [44–46]. When compared with other rates, MDD rates among Lebanese children in this study (45.3%) were lower than rates observed in the UAE (71.4%) [47], but higher than rates observed globally (29.4%) and in the MENA region (16%). In addition, rates of MDD among Syrian refugees’ children in this study (27.7%) were comparable with rates among refugees from Thailand-Myanmar borders (22.3%) [48]. Given the fragility of the situation, households participating in this study do not appear to have proper access to food and thus are expected to have such low MDD and MAD rates compared with other countries.

In this study, a few factors affected the odds of EBF at four and 6 months. Given the distinct breastfeeding profile of Syrian refugees and their host Lebanese communities, these factors were examined separately. Among Syrian refugees, the education level of the father and his employment status seemed to significantly influence EBF. More specifically, a higher educational level (intermediate school or above) among fathers was found to increase the odds of EBF at 4 months among Syrian refugee mothers compared with fathers whose educational level was primary school or less. Studies from both developed and developing countries such as Sweden and Bangladesh found similar results, where a higher educational level of parents contributed to better breastfeeding [49, 50]. In addition, having an employed spouse increased the odds of EBF for 4 months among Syrian refugee mothers. A husbands’ employment, especially for long hours, is very important and is often an overlooked way in which some spouses support and enable mothers to breastfeed, particularly as the entire family depend on the income of the spouse, and this was shown in a study done in the USA [51]. A husband’s employment often frees the mother from the burden of employment that can interfere with breastfeeding, especially EBF, as work schedule and stress can affect her ability to feed a baby.

As for the Lebanese host communities, the sex of the child and delivery mode were the factors found to significantly affect EBF for four and 6 months. The odds of EBF decreased when the gender of the child was female, indicating a possible preference of mothers to care for boys rather than girls. However, findings from another study in India showed that girls were exclusively breastfed more than boys. Interestingly, in the latter study, it seemed that mothers valued the health of boys more than girls, therefore they interrupted EBF earlier to increase intake of boys from other nutritious sources (solid foods) [52]. Taken together, the findings of this study and others underscored the need to account for gender when examining breastfeeding and complementary feeding practices. Furthermore, natural birth was found to increase the odds of EBF for 6 months among mothers of the Lebanese host communities. Such a finding was also observed in other studies in Lebanon, Jordan, Ethiopia, and Bangladesh where obstetric complications that may occur before and after Cesarean section are believed to interfere with breastfeeding, and the wound pain and anesthesia of the Cesarean section are thought to contribute to poor child nursing practices [40, 50, 53, 54].

Some limitations need to be considered when interpreting the results of this study. First, the inability to generalize the results to the whole Lebanese host and Syrian refugee communities, as Akkar represents a rural and impoverished area in Lebanon. However, the adequate sample size allows for the generalization of the data to similar communities. Another important limitation is that breastfeeding practices were self-reported, and the study is retrospective in nature and thus subject to social desirability and recall bias and misreporting. It should be noted however, that all interviews were conducted by trained dietitians who followed standardized techniques and procedures to ensure optimal collection of required data. Furthermore, it is noteworthy to indicate that the questionnaire used was not formally validated in the context of the study. That said, a panel of experts, consisting of a dietitian, a public health nutritionist and a nutrition epidemiologist, examined the face validity of the questionnaire. The content validity was further confirmed during the pilot-testing phase (described earlier in the Methods section). In addition, the cross-sectional nature of the study may suggest associations and does not establish causality.

## Conclusion

Optimal nutrition during the first 1000 days is particularly important as it can decrease the risk of chronic diseases, reduce morbidity and mortality, and promote better development overall. This study is the first of its kind to compare the breastfeeding and complementary feeding behaviors of Syrian refugees and their Lebanese host communities. However, despite the seemingly similar culture of the two study groups, Syrian refugees’ breastfeeding behavior was better with longer duration of breastfeeding and more EBF for four and 6 months compared with the Lebanese host community even though the latter fared somewhat better at the socio-economic level. Thus, the promotion of optimal infant and young child feeding practices in these communities can be part of health strategies incorporated at the primary public health level in order to improve the health, wellbeing, and nutrition of not only infants but also mothers.

The findings of this study highlight a need to adjust current breastfeeding education campaigns targeting Syrian refugee communities and initiate programs and campaigns among the Lebanese host communities, increasing the awareness of the benefits of breastfeeding and ensuring that obstacles that mothers may face during breastfeeding do not lead to breastfeeding cessation. Furthermore, the observed suboptimal complementary feeding behavior in both study groups suggests the need to develop specific educational programs to teach mothers when to introduce solid foods and what food items to initiate. These findings may be considered when planning future prospective studies that continue to investigate the feeding patterns among these vulnerable communities in order to assist in promoting the health and wellbeing of mothers and children.

## Data Availability

The datasets used and / or analyzed during the current study are available from the corresponding author on reasonable request.

## Abbreviations

BMI: Body Mass Index
CI: Confidence Interval
EBF: Exclusive breastfeeding
MAD: Minimum acceptable diet
MDD: Minimum dietary diversity
MENA: Middle East and North Africa
MMF: Minimum meal frequency
OR: Odds ratio
SD: Standard deviation
SPSS: Statistical Package for the Social Sciences
UAE: United Arab Emirates
UNHCR: United Nations High Commissioner for Refugees
USA: United States of America
WHO: World Health Organization
